# Surviving the Cretaceous-Paleogene mass extinction event: A terrestrial stem turtle in the Cenozoic of Laurasia

**DOI:** 10.1038/s41598-020-58511-8

**Published:** 2020-01-30

**Authors:** Adán Pérez-García

**Affiliations:** Grupo de Biología Evolutiva, Facultad de Ciencias, UNED, Paseo Senda del Rey 9, 28040 Madrid, Spain

**Keywords:** Palaeontology, Palaeontology

## Abstract

Findings of terrestrial stem turtles are not uncommon at Mesozoic continental sites in Laurasia, especially during the Upper Cretaceous. Thus, the record of several lineages is known in uppermost Cretaceous ecosystems in North America (Helochelydridae), Europe (Helochelydridae and *Kallokibotion*) and Asia (Sichuanchelyidae). No terrestrial stem turtle had been described in Laurasia after the Cretaceous-Paleogene mass extinction event. Thus, the only representatives described in the Cenozoic record worldwide corresponded to forms from southern Gondwana, where some of them survived until the Holocene. A bizarre terrestrial stem turtle from the upper Thanetian (upper Paleocene) of Europe is described here: *Laurasichersis relicta* gen. et sp. nov. Despite its discovery in France, in Mont de Berru (Marne), this Laurasian taxon is not recognized as a member of a European clade that survived the Cretaceous-Paleogene extinction event. It belongs to Sichuanchelyidae, a hitherto exclusively Asian Mesozoic group, known from the Middle Jurassic. Finds at the Belgian site of Hainin (Hainaut) show that this dispersion from Asia and the occupation of some niches previously dominated by European Mesozoic terrestrial stem forms had already taken place a few million years after the mass extinction event, at the end of the lower Paleocene.

## Introduction

The end of the Mesozoic involved a radical change in the diversity of many groups of terrestrial vertebrates, in which stem turtles (i.e., non-Testudines Testudinata) were not an exception. During the uppermost Cretaceous, three lineages of terrestrial stem turtles, all of them belonging to Perichelydia, inhabited Laurasia: the abundant and diverse helochelydrids, restricted to Euramerica, and known from the Late Jurassic (Tithonian); *Kallokibotion bajazidi*, from the Maastrichtian in Romania, the record of this exclusively European lineage being identified from the Santonian; and the Mongolian Maastrichtian *Mongolochelys efremovi*, recognized as the youngest descendant of an exclusively Asian clade, Sichuanchelyidae, identified from the Middle Jurassic, but with an extensive ghost lineage that spans from the Late Jurassic to the Late Cretaceous^1–5^. Therefore, they are all currently recognized as exclusively Mesozoic clades, and no stem terrestrial representative has been described in the Laurasian Cenozoic record. The only Cenozoic forms described worldwide correspond to a single clade, Meiolaniformes, exclusive to southern Gondwana, where it is known from the Early Cretaceous to the Holocene^6^.

The currently available information on the European Paleogene continental turtles shows that the faunal composition was radically different from that of the Maastrichtian^7–9^. In fact, most taxa identified in the Paleogene record in this continent are the result of new faunal dispersals, so that many of the niches previously occupied by Mesozoic turtles were populated by other lineages, from different continents^8^. Information on European Paleogene turtle diversity is very limited compared to that of other coeval records from elsewhere (especially the North American record^10^) and to those from the Maastrichtian and Eocene in Europe^7,9^. The presence of abundant remains of European Paleocene turtles was reported from the French upper Thanetian (upper Paleocene) area of Mont de Berru (Marne)^8,11^. The existence of an undetermined genus in Mont de Berru that could be close to the Cretaceous *Kallokibotion* was reported more than 40 years ago^11^. Information on the anatomy and diversity of the Laurasian Cretaceous stem Testudines was then extremely limited, as only the skull of *Kallokibotion bajazidi* was partially known. Although the presence of a turtle from Mont de Berru with several character states shared with forms currently considered as belonging to the stem group was also supported in subsequent papers^12,13^, no figure or description was provided. Therefore, more recent publications indicated that, based on the very scarce information provided on that French form, it is not possible to verify that claim, which cannot be substantiated^3^. The confirmation of this hypothesis could have relevant implications because it would imply the existence of a lineage of Laurasian stem Testudines that survived the Cretaceous-Cenozoic extinction event^3^.

Knowledge about the Laurasian Cretaceous terrestrial stem Testudines has improved dramatically during the last decade. The cranial anatomy of the helochelydrids has been described for the first time, from North American and European forms; knowledge of the postcranial skeleton of this group has also markedly increased^1,5,14^; a detailed study on the so far poorly known European *Kallokibotion bajazidi* has recently been performed^4^; and the enigmatic *Mongolochelys efremovi* is now much better understood after being recognized as belonging to the so far exclusively Asian clade Sichuanchelyidae, with knowledge about this lineage also increasing^2^. Improvement in the knowledge of these lineages, recognized as exclusive to Laurasia^2–5^, offers the appropriate framework to carry out here the detailed study of the aforementioned European Paleocene turtle, represented by numerous remains. Its attribution to a terrestrial member of the stem group of Testudines can be confirmed. The phylogenetic and paleobiogeographic origin of this new and peculiar taxon, which evidences the presence of a stem lineage of turtles as a relict in the Cenozoic record, is discussed. In fact, it cannot be recognized as the survivor of a clade present in the European Mesozoic record. Its presence is identified as the result of dispersal from outside the continent, which resulted in the occupation of a niche previously dominated by European Mesozoic lineages.

## Systematic paleontology

Testudinata Klein, 1760

Mesochelydia Joyce, 2017

Perichelydia Joyce, 2017

Sichuanchelyidae Tong *et al*., 2012

*Laurasichersis relicta* gen. et sp. nov.

(Figures 1–7)

**Figure 1:**
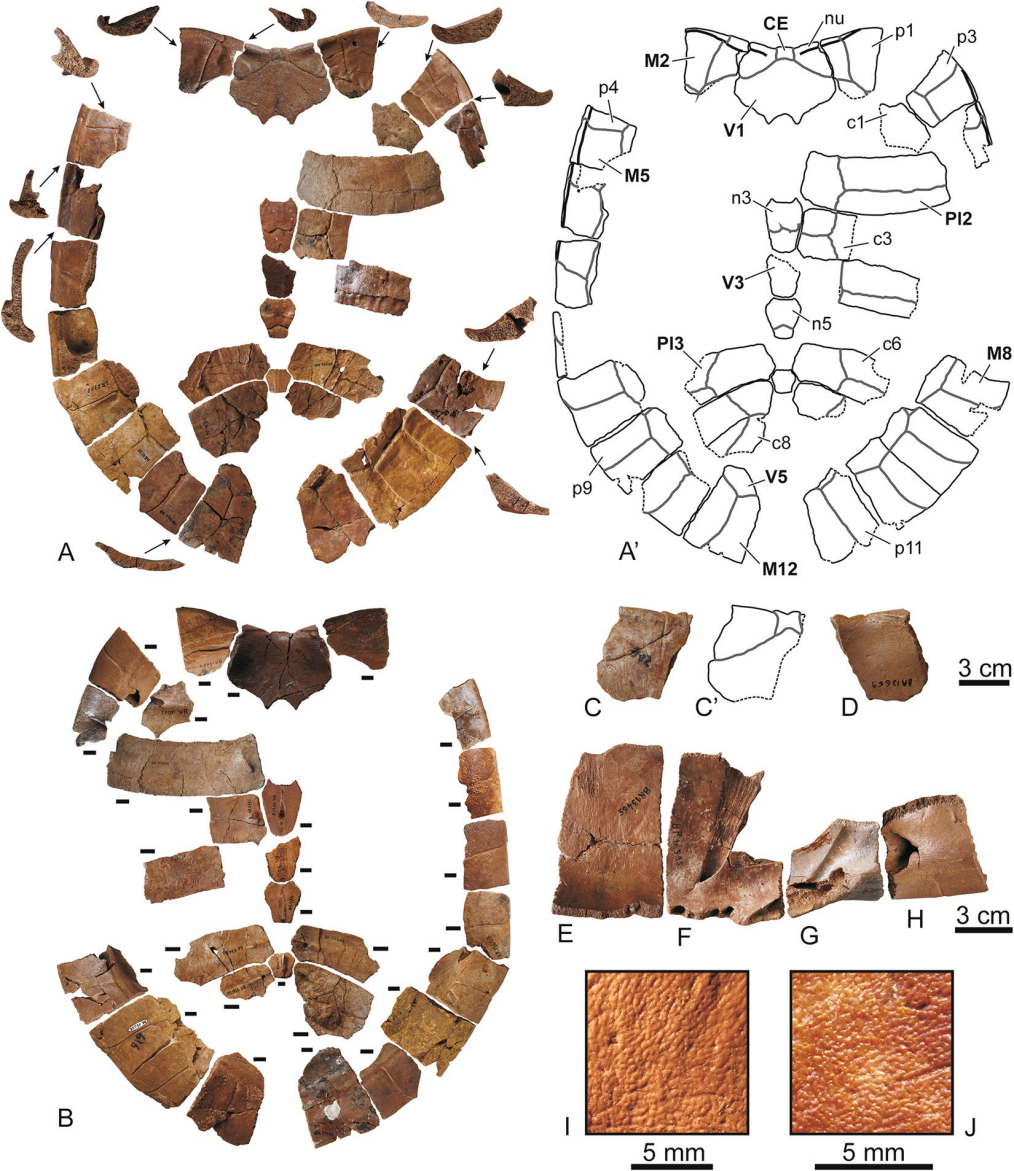
Elements of the carapace of the stem turtle (Sichuanchelyidae) *Laurasichersis relicta* gen. et sp. nov., from the upper Thanetian (upper Paleocene) of Mont de Berru (Marne, France). (**A**,**B**), carapace plates corresponding to several individuals, in dorsal (**A**) and ventral (**B**) views: nuchal MNHN.F BR13461; neurals (anterior to posterior) MNHN.F BR13458, MNHN.F BR17487, MNHN.F BR13710, MNHN.F BR13459; right costals (anterior to posterior) MNHN.F BR13001, MNHN.F BR13620, MNHN.F BR2785, MNHN.F BR18001, MNHN.F BR13637, MNHN.F BR13628; left costals (anterior to posterior) MNHN.F BR13624, MNHN.F BR4188; right peripherals (anterior to posterior) MNHN.F BR13604, MNHN.F BR18000, MNHN.F BR13611, MNHN.F BR13468, MNHN.F BR15098, MNHN.F BR13610; left peripherals (anterior to posterior) MNHN.F BR13603, MNHN.F BR18002, MNHN.F BR13595, MNHN.F BR13465, MNHN.F BR13608, MNHN.F BR2758, MNHN.F BR4180, MNHN.F BR13480, MNHN.F BR13467. The anterior or posterior views of the peripherals are also shown in A. Scale bars equal 1 cm. For the identification of each element see Fig. 4A. (**C**,**D**), nuchal MNHN.F BR13669, in dorsal (**C**) and ventral (**D**) views. (**E–H**), visceral view of the the third (right) to the sixth (left) bridge peripherals MNHN.F BR13622, MNHN.F BR13623, MNHN.F BR18002 and MNHN.F BR18000. (**I**,**J**), details of the outer surface of the neural MNHN.F BR13459 (**I**) and costal MNHN.F BR13579 (**J**). Abbreviations for the plates (in lowercase and normal type): c, costal; n, neural; nu, nuchal; p, peripheral. Abbreviations for the scutes (in uppercase and in bold type): CE, cervical; M, marginal; PL, pleural; V, vertebral.

**Figure 2:**
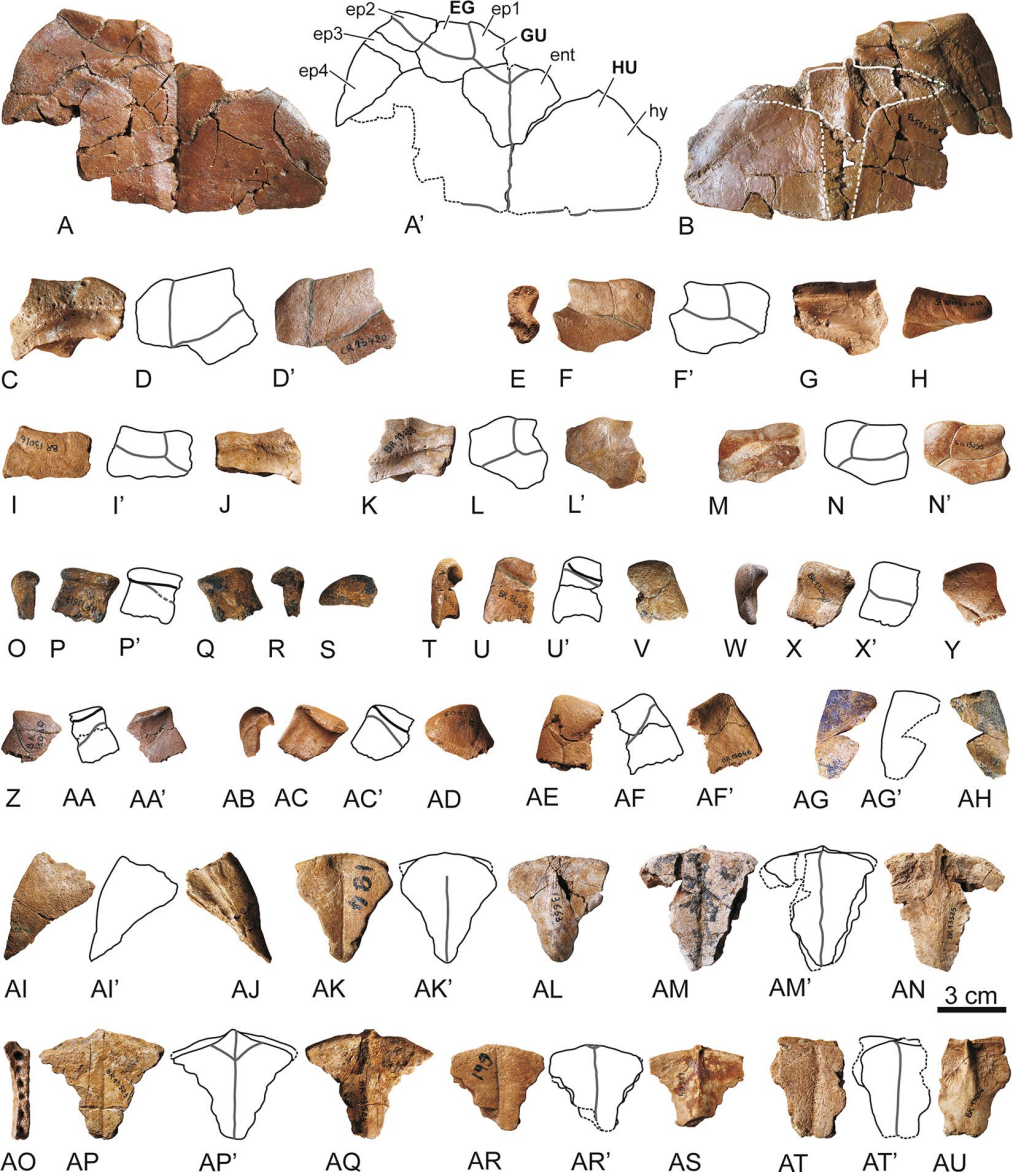
Elements of the anterior plastral lobe of the stem turtle (Sichuanchelyidae) *Laurasichersis relicta* gen. et sp. nov., from the upper Thanetian (upper Paleocene) of Mont de Berru (Marne, France). (**A**,**B**), MNHN.F BR13513, holotype of the taxon, articulated right epiplastral elements, entoplastron, and anterior regions of both hyoplastra, in ventral (**A**) and dorsal (**B**) views. The visceral position of the entoplastron margins are indicated in B. C–AU, medial (**C–N**): MNHN.F CR13420, MNHN.F BR13005, MNHN.F BR13016, MNHN.F BR13058, MNHN.F CR13055), antero-lateral and lateral (O–AF: MNHN.F BR13617, MNHN.F BR13063, MNHN.F BR13068, MNHN.F BR13010, MNHN.F BR13009, MNHN.F BR15046), and postero-lateral (AG–AJ: MNHN.F BR17488, MNHN.F BR17489) epiplastral elements. Al of them in dorsal and ventral views, but some elements also in lateral (E, O, T, W, AB), medial (R) and anterior (H, P) ones. AK–AU, entoplastra, MNHN.F BR13663 (AK–AL), MNHN.F BR13525 (AM–AN), MNHN.F BR13523 (AO–AQ), MNHN.F BR13664 (AR–AS) and MNHN.F BR13506(AT–AU); in ventral and dorsal views. Abbreviations for the plates (in lowercase and normal type): ent, entoplastron; ep, epiplastral element; hy, hyoplastron. Abbreviations for the scutes (in uppercase and in bold type): EG, extragular; GU, gular; HU, humeral.

**Figure 3:**
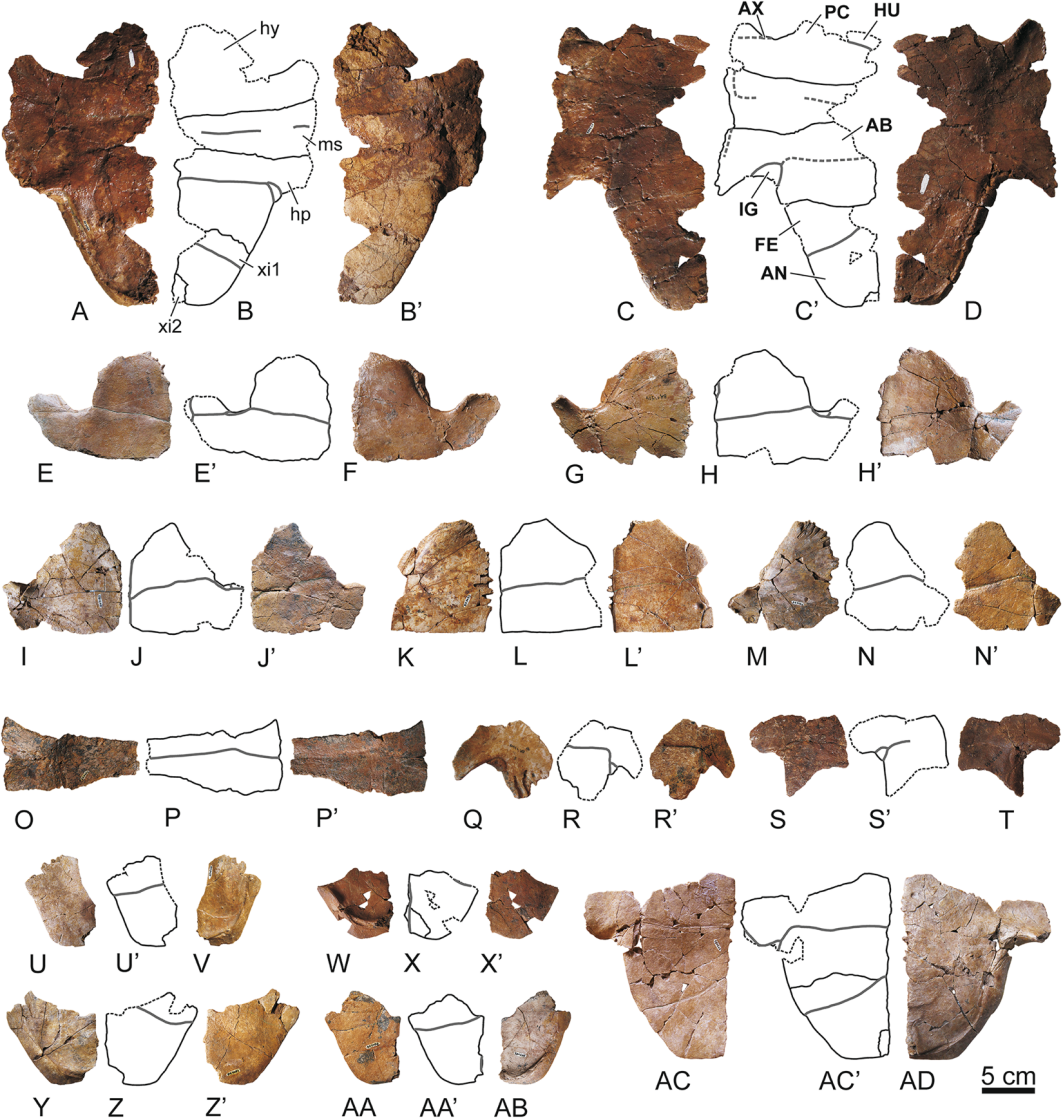
Elements of the plastron of the stem turtle (Sichuanchelyidae) *Laurasichersis relicta* gen. et sp. nov., from the upper Thanetian (upper Paleocene) of Mont de Berru (Marne, France). (**A–D**), two articulated partial hemiplastra (MNHN.F BR9069, MNHN.F BR9068), including most of the hyoplastra, mesoplastra, hypoplastra and xiphiplastral elements. (**E–N**), hyoplastra (MNHN.F BR13515, MNHN.F BR13514, MNHN.F BR9085, MNHN.F BR9033, MNHN.F BR9084). (**O**,**P)**, mesoplastron (MNHN.F BR9070). (**Q–T**), hypoplastra (MNHN.F BR13508, MNHN.F BR13491). U–AB, xiphiplastral elements (MNHN.F BR9079, MNHN.F BR17486, MNHN.F BR9081, MNHN.F BR9089). AC–AD, articulated right hypoplastron and xiphiplastra (MNHN.F BR9083). All of them in ventral and dorsal views. Abbreviations for the plates (in lowercase and normal type): hp, hypoplastron; hy, hyoplastron; ms, mesoplastron; xi, xiphiplastral element. Abbreviations for the scutes (in uppercase and in bold type): AB, abdominal; AN, anal; AX, axillar; FE, femoral; HU, humeral; IG, inguinal; PC, pectoral.

**Figure 4:**
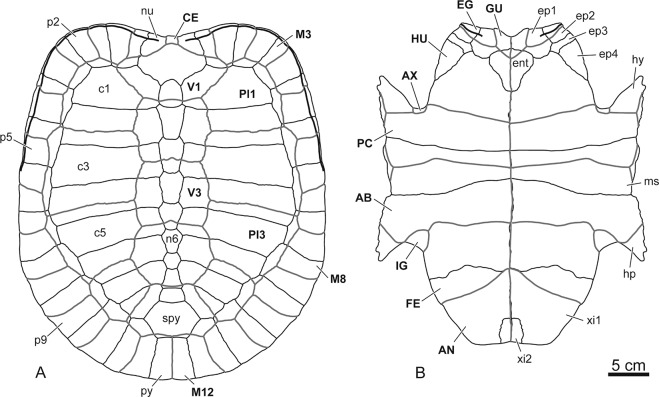
Reconstruction of the shell of stem turtle (Sichuanchelyidae) *Laurasichersis relicta* gen. et sp. nov., from the upper Thanetian (upper Paleocene) of Mont de Berru (Marne, France). (**A**), dorsal view of the carapace. (**B**), ventral view of the plastron. Abbreviations for the plates (in lowercase and normal type): c, costal; ent, entoplastron; ep, epiplastral element; hp, hypoplastron; hy, hyoplastron; ms, mesoplastron; n, neural; nu, nuchal; p, peripheral; py, pygal; spy, suprapygal; xi, xiphiplastral element. Abbreviations for the scutes (in uppercase and in bold type): AB, abdominal; AN, anal; AX, axillar; CE, cervical; EG, extragular; FE, femoral; GU, gular; HU, humeral; IG, inguinal; M, marginal; PC, pectoral; PL, pleural; V, vertebral.

**Figure 5:**
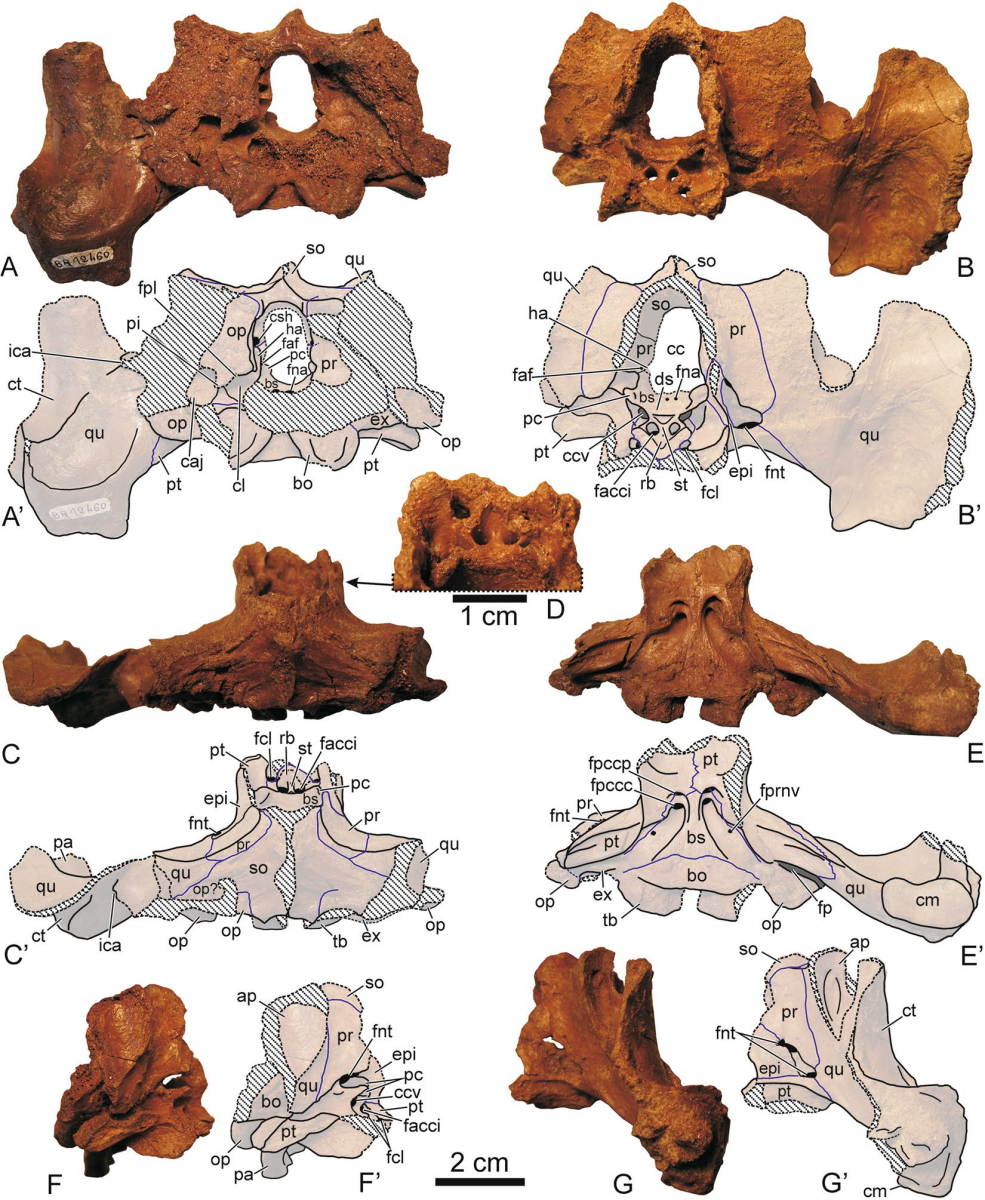
MNHN.F BR12460, partial skull of the stem turtle (Sichuanchelyidae) *Laurasichersis relicta* gen. et sp. nov., from the upper Thanetian (upper Paleocene) of Mont de Berru (Marne, France), corresponding to its paratype, in posterior (**A**), anterior (**B**), dorsal (**C,D**), ventral (**E**), right lateral (**F**) and left lateral (**G**) views. Abbreviations: ap, antrum postoticum; bo, basioccipital; bs, basisphenoid; caj, cavum acustico-jugulare; cc, cavum cranii; ccv, canalis cavernosus; cl, cavum labyrinthicum; cm, condylus mandibularis; csh, canalis semicircularis horizontalis; ct, cavum tympani; ds, dorsum sellae; epi, epipterygoid; ex, exoccipital; facci, foramen anterius canalis carotici interni; faf, fossa acustico-facialis; fcl, foramen caroticum laterale; fna, foramen nervi abducentis; fnt, foramen nervi trigemini; fp, fenestra postotica; fpccc: foramen posterius canalis carotici cerebralis; fpccp: foramen posterius canalis carotici palatinum; fpl, fenestra perilymphatica; fprnv, foramen pro ramo nervi vidiani; ha, hiatus acusticus; ica, incisura columella auris; op, opisthotic; pa, processus articularis; pc, processus clinoideus; pi, processus interfenestralis; pr, prootic; pt, pterygoig; qu, quadrate; rb, rostrum basisphenoidale; so, supraoccipital; st, sella turcica; tb, tuberculum basioccipitale.

**Figure 6:**
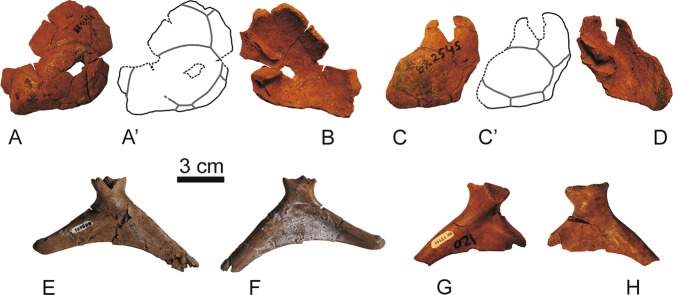
Right **s**quamosals (**A**,**B**), MNHN.F BR13358; (**C**,**D**), MNHN.F BR2545) and left and rigth scapulae (**E**,**F**), MNHN.F BR9051; (**G**,**H**), MNHN.F BR15111) of the stem turtle (Sichuanchelyidae) *Laurasichersis relicta* gen. et sp. nov., from the upper Thanetian (upper Paleocene) of Mont de Berru (Marne, France). Squamosals in dorsal and ventral views. Scapulae in anterior and posterior views.

**Figure 7:**
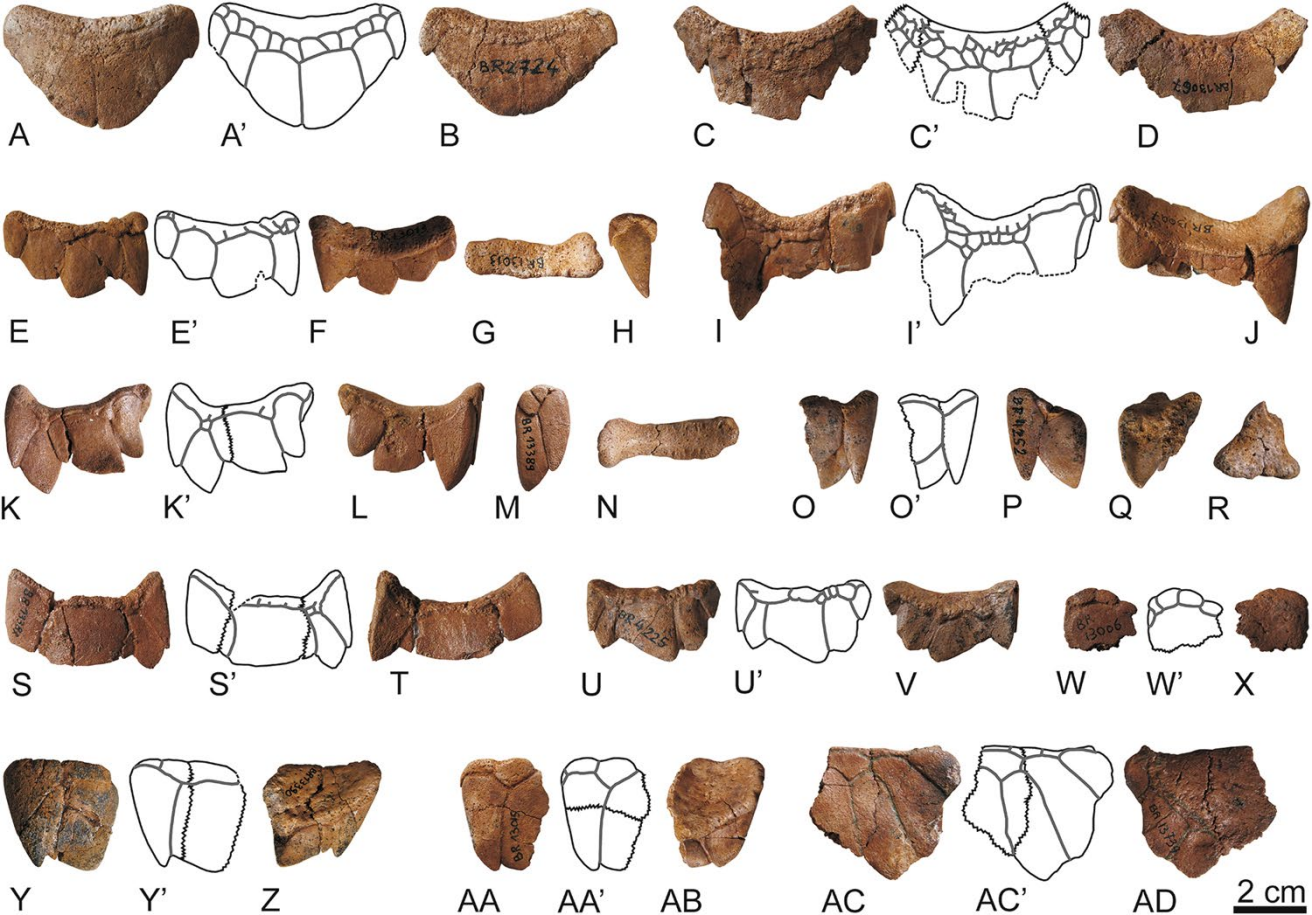
Osteoderms of the stem turtle (Sichuanchelyidae) *Laurasichersis relicta* gen. et sp. nov., from the upper Thanetian (upper Paleocene) of Mont de Berru (Marne, France). (**A**,**B**), MNHN.F BR2724; (**C,D**), MNHN.F BR13067; (**E–H**), MNHN.F BR13013; (**I**,**J**), MNHN.F BR13007; (**K–N**), MNHN.F BR13389; (**O–R**), MNHN.F BR4252; (**S,T**), MNHN.F BR13387; (**U**,**V**), MNHN.F BR4225; (**W**,**X**), MNHN.F BR13006; (**Y**,**Z**), MNHN.F BR13390; AA–AB, MNHN.F BR13015; AC–AD, MNHN.F BR13739.

### Holotype

MNHN.F BR13513, partial anterior plastral lobe, including the articulated right epiplastral elements, entoplastron, and anterior regions of both hyoplastra (Fig. 2A,B).

### Paratype

MNHN.F BR12460, partial posterior half of a skull (Fig. 5).

### Referred specimens

Selection of 77 specimens from the type locality and horizon, represented in Figs. 1, 2E–L, 2O–AU, 3, 6, 7. They correspond to articulated and disarticulated plates (Fig. 1, 2C–AU, 3), two squamosals (Fig. 6A–D), two scapulae (Fig. 6E–H), and several osteoderms (Fig. 7). Two medial epiplastral elements in Fig. 2C,D and 2M–N from the ‘Conglomérat de Cernay’ (MP6a, upper Thanetian) of Lemoine quarry (Mont de Berru, Berru, France)^15^.

### Locality and Horizon

Mouras quarry, Mont de Berru, Berru, Marne, France. Sables de Bracheux Formation, Franco-Belgian Basin. MP6a, upper Thanetian, upper Paleocene^8,16^.

### Etymology

The generic name is composed by Laurasi-, referring to Laurasia, where this taxon comes from; and –chersis, a Latinized word of Greek origin (Χέρσος) which means “land or dry land”, following the criteria used to establish the generic names of some other terrestrial stem turtles^5^. The specific name refers to the fact that the new taxon is a vestige of Mesozoic fauna, being the only known Laurasian post-Mesozoic terrestrial stem turtle.

### Diagnosis

Sichuanchelyid defined by the following characters exclusive within this clade: dorsally directed distal margin of the anterior and middle peripherals, and antero-lateral margins of the nuchal; slightly wider than long nuchal; high bridge peripherals; second to fourth vertebrals narrower than the first one, and than the second and third pleurals; concave anterior plastral margin; epiplastra divided into four elements, acquiring an exclusive morphology; supernumerary xiphiplastra, divided into two elements; humeral-pectoral sulcus at the level of the axillary notch; absence of a complete inframarginal series, but presence of axillar and inguinal scutes; anterior and posterior pairs of ventral foramina for the carotids closer to each other than between the foramina that form each pair. This sichuanchelyid shows the following unique character combination: shell size greater than 60 cm; second costal as long as the first; absence of contact and long distance between the nuchal postero-lateral end and the second peripherals; first vertebral wider than the nuchal; contact of the first vertebral with the second marginals; pleural-marginal sulci on the proximal region of the peripherals; anterior end of the axillary buttress reaching the anterior half of the third peripherals; absence of plastral fontanelles; absence of strongly interfingered plastral contacts; supernumerary epiplastra; long epiplastral symphysis relative to the entoplastron length; gulars overlapping the antero-medial entoplastral area; wide exposure of the squamosals on the skull table; absence of palatal teeth; absence of ventral exposure of the prootics; narrow and deep depression between the tubercula basioccipitale; short basicranium in relation to its width; absence of cleithrum processes.

### Description

The shell can acquire a length greater than 60 cm. It lacks fontanelles. The carapace is longer than it is wide (Fig. 1A,B). It shows a very wide anterior notch, which develops over the anterior margins of the nuchal and the complete first peripherals. The carapace lateral margins are subparallel. Although the bridge peripherals are high, the costals do not generate a very high carapace. The posterior peripherals are long. The distal margins of some of the anterior and medial peripherals are markedly guttered, and the antero-lateral margins of the nuchal are also dorsally directed in some specimens. The outer surface of the shell is finely granulated (Fig. 1J,K). Well-developed growth rings are identified on some costals (Fig. 1A,B).

The nuchal is slightly wider than it is long (Fig. 1A–D). The neurals contact this plate, but also the suprapygal series. Most preserved neurals are recognized as hexagonal, longer than they are wide, except the last ones, which are as wide as long, and the first one, interpreted as subrectangular. The length of the first costal is close to that of the second. This taxon has a single cervical, almost as long as wide. It has five vertebrals, all of them wider than they are long. The first is the widest, being wider than the nuchal, and contacting the second marginals. The second to fourth vertebrals are narrower than the first to third pleurals. This turtle lacks supramarginals. The marginals are restricted to the peripherals, but reach a position close to the suture of these plates with the costals. The last pair of marginals reaches the suprapygal series.

The connection between the carapace and the plastron is partially ligamentous (Figs. 1A,B,E–H and 3A–D). The plastral buttresses do not contact the costals. The anterior ones penetrate a cavity located on the posterior half of the third peripherals, reaching the anterior half of these plates. The inguinals are inserted into depressions located on the anterior half of the eighth peripherals. The plastron is long relative to the carapace length (Fig. 4). Its anterior margin probably reached a position close to that of the carapace. The bridge is almost as long as the posterior plastral lobe, which is longer than the anterior one (Fig. 3A–D). Both plastral lobes are wide. The anterior is trapezoidal, with a slightly concave anterior margin, especially in the region corresponding to the gulars, where a notch is medially located (Fig. 2A–N). Each epiplastron is divided into four osseous elements (Fig. 2A–AJ). The medial is the largest. The most posterior one is elongated, so that the epiplastra reach more than half of the lateral lobe length. The contact of these elements with the entoplastron and with the hyoplastra was ligamentous, this union corresponding to a long groove in the epiplastra. A well-developed dorsal thickening is present in the anterior margin of the epiplastra. In ventral view, the entoplastron varies from almost as wide as long to longer than wide, being subtriangular, with a substraight anterior margin (Fig. 2A,AK–AU). Viscerally, it shows long posterior and lateral processes (Fig. 2B). Its contact with the hyoplastra is ligamentous, with aligned marked depressions for the insertion of small hyoplastral spikes (Figs. 2AO, 3E,M). A pair of mesoplastra, contacting each other in the medial plane, is present (Fig. 3A–D,O–P). Each of the xiphiplastra is formed by a pair of osseous elements, one of them, much smaller, being postero-medially located (Fig. 3A–D,U–AD). The morphology of these small elements is variable. *Laurasichersis relicta* lacks an anal notch. A pair of gulars is present, reaching the most anterior region of the entoplastron (Fig. 2A–N). Most extragulars are far from the entoplastron (Fig. 2A,F,I,L,N), but specimens in which they contact the antero-lateral margin of this plate are known (Fig. 2D). The humero-pectoral sulci are markedly far behind the entoplastron, at the level of the axillary notches (Figs. 2A,3E–N). This taxon lacks a continuous inframarginal series, but axillary and inguinal scutes are present (Figs. 3, 4B). The anals are placed on the xiphiplastra, medially reaching the hypoplastra.

Two regions of the skull are identified. One of them corresponds to the articulated bones of the partial posterior half of a skull (i.e., the otic chambers, occiput, and braincase), lacking the dermal roofing elements (Fig. 5). Considering its dimensions, the total width of this skull, at the posterior area, was about 13 cm. The basicranium is short in relation to its width. The pterygoids show a well-developed medial contact, an open interpterygoid vacuity being absent (Fig. 5E). No palatal teeth are developed, at least in the preserved region. A pair of wide pits for the carotids branches is present in the ventral surface of the basisphenoid. Both are close to the medial plane, but the distance between these pairs is smaller than that between each of the foramina that form each pair. The most anterior pair is located on the limit between the basisphenoid and the pterygoids. These foramina have a slightly larger diameter than the posterior ones. The posterior foramina are located at the end of a pair of deep and well developed grooves, which run along the lateral regions of the basisphenoid, and are postero-laterally oriented. In the cavum cranii, the foramina caroticum laterale and the foramina anterius canalis carotici interni retain a wide diameter, also being close to each other (Fig. 5B–D). The rostrum basisphenoidale is almost flat. In ventral view, the basisphenoid is triangular and wider than long (Fig. 5E). This taxon lacks a ventral exposure of the prootics. A narrow and deep depression is developed between both tubercula basioccipitale. A quadrate-basisphenoid contact is absent, as well as the contact between the pterygoids and the basioccipital. The mandibular condyles of the quadrates are twice as wide as they are long. They are located in a more posterior position in relation to the foramina posterius canalis carotici cerebralis. In lateral view, the antrum postoticum and the cavum tympani are interpreted as tall in relation to their length, which is compatible with the recognition of the basicranium as relatively short (Fig. 5F,G).

The other preserved elements of the skull are several squamosals (Fig. 6A–D). They display a large dorsal exposure, so that the skull roof of *L. relicta* lacked a temporal emargination. The posterior margins of these bones were well posterior to the antrum postotic (Fig. 6B,D). In the skull roof, these bones were better developed in width than in length. Small protuberances are developed on their posterolateral region (Fig. 6A–C). The squamosals were covered by several well-developed scutes, including a central one and several adjacent ones, some of them reaching the posterior margin of the skull roof and others overlapping the adjacent bones.

*Laurasichersis relicta* lacks processes corresponding to the cleithrum (Fig. 2B). The shoulder girdle is a triradiate element (Fig. 6E–H). The neck of the glenoid is well-developed. The scapular and acromial processes of the scapula are rod-shaped elements, elliptical in section. A well-developed lamina is present between both processes.

This taxon has osteoderms (Fig. 7). Some of them consist of a single osseous element (Fig. 7A,B,E–J,U–V), but others by several, sutured between them (Fig. 7C,D,K–T, W–AD). As is common for these elements, their morphology is diverse, depending on their anatomical position. Each of these osteoderms was covered by several scutes.

## Discussion

**Figure 8:**
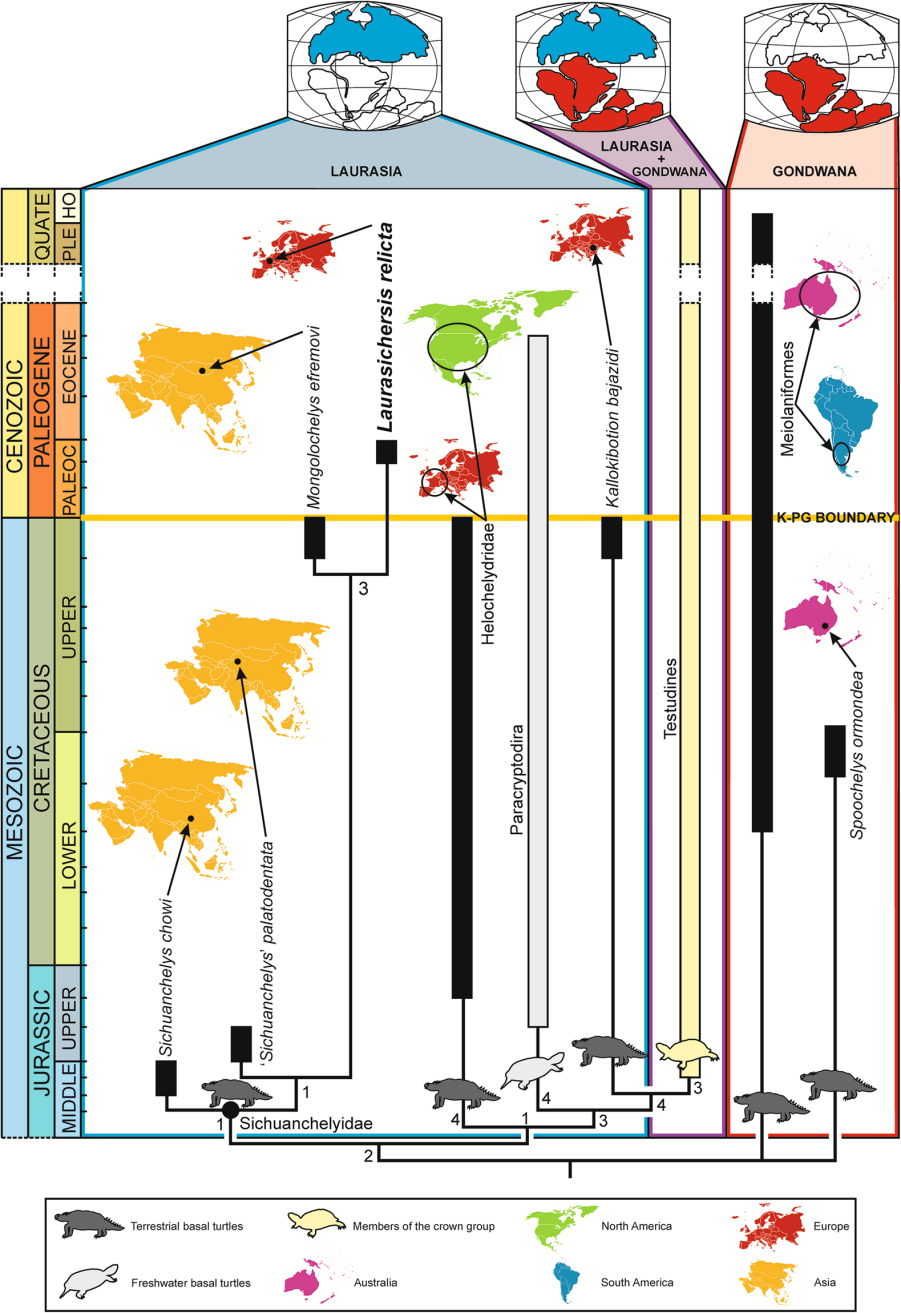
Calibrated cladogram corresponding to the cladistic analysis performed here (4500 most parsimonious trees, length of 943 steps, CI = 0.316, RI = 0.778, RC = 0.246), in which the position of all representatives of Perichelydia (sensu^3^) is shown, as well as that of the crown group Testudines. This distribution is based on the strict consensus tree, except in the case of the clade Meiolaniformes, which is obtained in the majority rule tree (67%). The paleobiogeographic distribution of each taxon is indicated. Bremer support values are shown.

Although, as obtained by the cladistic analysis performed here (Fig. 8), the European *Laurasichersis relicta* and *Kallokibotion bajazidi* correspond to two terrestrial stem turtles with a phylogenetic position close to that of the crown group Testudines (i.e., representatives of Perichelydia), the shell of the new Paleocene species markedly differs from that of the uppermost Cretaceous taxon in numerous characters, including several that show more primitive states in the new form (i.e., states shared with members of Mesochelydia): absence of the *K. bajazidi* autapomorphic nuchal, formed by two elements; presence of a nuchal notch; presence of dorsally directed distal margins of the anterior and medial peripherals; presence of a cervical; absence of contact of the plastral buttresses with the costals; absence of an osseous carapace-plastron connection; absence of a rounded anterior plastral lobe; presence of a marked epiplastral lateral development, which extends behind the level of contact of these plates with the entoplastron; absence of a subrounded to subrhombic entoplastron, almost as long as wide; presence of posterior and lateral entoplastral processes; absence of an anal notch; presence of a long separation between the humeral-pectoral sulcus and the entoplastron^4^. Some of those shell characters are also radically different from those of the other lineage of stem turtles identified in the European record: Helochelydridae. Thus, the representatives of this clade do not share with *L. relicta* the presence of a well-developed postero-lateral development of the epiplastra; long lateral entoplastral processes; and long separation between the humero-pectoral sulcus and the entoplastron. It addition, *L. relicta* differs from the helochelydrids by the absence of a complete inframarginal series, and of three synapomorphies of this clade: outer surface of the shell covered by well-developed tubercles; visceral cavity of the peripherals not restricted to the bridge area; and development of an entoplastral scute^1,5^. Therefore, the shell of *L. relicta* demonstrates that this new form cannot be attributed to Helochelydridae, but neither identified as a form closely related to *K. bajazidi*. By contrast, all these shell characters are shared with the taxon from the Maastrichtian of Mongolia *Mongolochelys efremovi*. In fact, *L. relicta* shares a synapomorphy of that clade^2,3^ not only with that species but also with the other members of the Asian clade Sichuanchelyidae (i.e., the Middle Jurassic *Sichuanchelys chowi* and the Upper Jurassic ‘*Sichuanchelys*’ *palatodentata*) a synapomorphy of that clade:^2,3^ the development of a very wide nuchal notch, delimited by the second peripherals. In addition, it shares all shell characters included in the exclusive combination proposed for this clade:^2,3^ presence of wider than long vertebrals, marginals restricted to the peripherals, broad plastron, short epiplastral symphysis in relation to the total length of these plates, and presence of a pair of fully developed mesoplastra. In fact, the cladistic analysis performed here supports the position of *L. relicta* as a member of the hitherto exclusively Asian clade Sichuanchelyidae and, in particular, as the sister taxon of *M. efremovi* (Fig. 8).

*Laurasichersis relicta* share several shell characters with *M. efremovi* but not with one or the other two members of Sichuanchelyidae^2,17–20^, revealing an exclusive combination with this taxon: shell size greater than 60 cm (whereas it is less than 20 cm in *S. chowi*, and less than 40 cm in ‘*S*.’ *palatodentata*); second costal as long as the first (in contrast to *S. chowi*, in which it is noticeably shorter); marginals restricted to the peripherals, but medially close to the costal-peripheral sutures (in contrast to *S. chowi*, in which they are noticeably narrower); supernumerary epiplastral plates (exclusive to *L. relicta* and *M. efremovi* within Testudinata); transversely oriented anterior plastral margin (in contrast to the other sichuanchelyids); longer epiplastral symphysis than in the other sichuanchelyids. The shell of *L. relicta* differs from that of *M. efremovi* in: higher carapace instead of markedly flattened (and higher than in the other sichuanchelyids); slightly wider than long nuchal instead of twice as wide as long (being longer than in the other sichuanchelyids); absence of contact and development of a longer distance between the nuchal postero-lateral margin and the second peripherals (shared with the other sichuanchelyids); dorsally directed distal margins of the anterior and middle peripherals, and antero-lateral margins of the nuchal (in contrast to all other sichuanchelyids); first vertebral wider than the nuchal (shared with the other sichuanchelyids), contacting the second pair of marginals (shared with ‘*S*.’ *palatodentata* but not with *S. chowi*); second to fourth vertebrals narrower than the first one and than the second and third pleurals (in contrast to all other sichuanchelyids); anterior end of the axillary buttress reaching the anterior half of the third peripherals rather than the margin between these peripherals and the second pair, penetrating in a well-developed osseous depression, whose margins are located in the posterior half of the third peripherals (unknown for the other sichuanchelyids); absence of plastral fontanelles (a central and a posterior plastral fontanelles probably also developed in the adults of ‘*S*.’ *palatodentata* but not in *S. chowi*); concave anterior plastral margin, from the latero-anterior tips to the medial area (in contrast to all other sichuanchelyids); greater number of supernumerary epiplastral elements, each epiplastra being divided into four instead of two, and acquiring an exclusive morphology for this taxon; absence of strongly interfingered plastral contacts (shared with *S. chowi* but not with ‘*S*.’ *palatodentata*), including those of the autapomorphic hyoplastra of *M. efremovi*, which overlap much of the entoplastral ventral surface, and the postero-ventral area of the epiplastra; autapomorphic supernumerary xiphiplastra; gulars overlapping the antero-medial entoplastral region (shared with the other sichuanchelyids); absence of a complete inframarginal series (not shared with *S. chowi*, which possesses four inframarginals, this character being unknown for ‘*S*.’ *palatodentata*), but presence of axillar and inguinal scutes; humeral-pectoral sulcus placed at the axillary notch level (in contrast to all other sichuanchelyids).

Since *L. relicta* corresponds to a member of Perichelydia, its skull lacks an open interpterygoid vacuity^3^. It differs radically from the helochelydrids, characterized by the outer surface of the skull covered by distinct tubercles and the presence of a secondary pair of occipital tubercles formed by the pterygoids^1,5,14^. In addition, the presence of paired pits on the ventral surface of the basisphenoid, for the carotids branches, is not shared with the helochelydrids, but with both *K. bajazidi* and the sichuanchelyids in which the skull is known (‘*S*.’ *palatodentata* and *M. efremovi*)^2,4,12,17–19^. In contrast to *K. bajazidi*, the foramina that form the posterior pair in *L. relicta* do not show a greater separation from each other than the anterior ones, but slightly smaller, this character being shared with the sichuanchelyids. Both pairs of foramina are much closer to each other in *L. relicta* than in the other sichuanchelyids and in *K. bajazidi*. The presence of a narrow and deep depression between the tubercula basioccipitale is shared with *M. efremovi*, but not with ‘*S*.’ *palatodentata* or with *K. bajazidi*. The preserved region of the *Laurasichersis relicta* skull lacks the palatal teeth developed on the medial surface of the pterygoids of ‘*S*.’ *palatodentata*. As with the shell, the large size of the skull analyzed here is only shared with *M. efremovi*. *Laurasichersis relicta* also shares with this form, but not with the other two taxa, the wide exposure of the squamosals on the skull table, in relation to their antero-posterior development. Both taxa share the presence of small protuberances on the posterolateral region of these bones. The absence of ventral exposure of the prootics of the new sichuanchelyid studied here is shared with *M. efremovi*, but contrasts with the condition in ‘*S*.’ *palatodentata*. The pattern of the scutes on the cranial table of ‘*S*.’ *palatodentata* is unknown. The pattern in *K. bajazidi* differs radically from that of *L. relicta*, not only in the morphology of the scutes but in that those of the Paleocene taxon are much less numerous, at least in the region corresponding to the squamosals. The presence of several scutes on the squamosals is shared with *M. efremovi*, both possessing a central scute and several adjacent ones, which reach the posterior margin of the skull or overlap the adjacent bones. The mandibular condyles of *L. relicta* are located in a more posterior position in relation to the foramina posterius canalis carotici cerebralis than those of *M. efremovi* and ‘*S*.’ *palatodentata*. Its basicranium is shorter in relation to its width that that of *M. efremovi*, as in ‘*S*.’ *palatodentata*. Its skull was probably taller than those of both taxa.

Although a reduced process corresponding to the cleithrum is present in *M. efremovi*, this structure has been completely lost in *L. relicta*, as was also the case in *K. bajazidi* and Helochelydridae. The presence of a well-developed lamina between the dorsal and the acromial processes of the scapula, shared with the members of Sichuanchelyidae, contrasts with the condition in *K. bajazidi*, in which it is markedly reduced^4,12^. An intermediate degree of development is known for the members of Helochelydridae^1,5,14^. The presence of osteoderms allows the identification of *L. relicta* as a terrestrial form. These structures are identified in several groups of terrestrial turtles, including forms of both the stem (e.g., Meiolaniformes, Helochelydridae) and the crown group (e.g., Testudinidae). The hitherto known record of Sichuanchelyidae had not allowed the presence of osteoderms in this lineage to be determined and they have been recognized here for the first time. The presence of osteoderms *in K. bajazidi* is unknown. The osteoderms of *L. relicta*, formed by different elements sutured together, are exclusive to this turtle. Only a few of the so far known osteoderms of the helochelydrids were covered by several scutes^13^.

## Implications and Conclusions

All clades of Cretaceous terrestrial stem turtles were until now regarded as restricted throughout their evolutionary history to the land areas upon which they originated^2^. However, the analysis of the new taxon studied here has identified a dispersion event for Sichuanchelyidae, from Asia to Europe. Thus, after the currently recognized disappearance of the terrestrial stem turtles known in the Upper Cretaceous in Europe (*Kallokibotion bajazidi* and several members of Helochelydridae^4,13^) at the end of the Maastrichtian, probably as a result of the Cretaceous-Paleogene mass extinction event, some of the available European niches are identified here as occupied by this Asian lineage.

The analysis of the new form from the upper Thanetian (upper Paleocene, MP6a) in France allows the reassignment of some remains from the upper Danian (lower Paleocene, MP2 or MP3) site of Hainin (Province of Hainaut)^16^, also located in the Franco-Belgian Basin, but in Belgium. The presence of several turtle taxa was recognized in that locality^21,22^, one of them being attributed to an indeterminate member of Baenidae^21^, and subsequently to cf. Baenoidea^22^. These attributions cannot be currently supported^8^. The number of elements of that turtle so far known is limited^21,22^, but they provide information that allows its systematic attribution to be evaluated here. The size and ornamental pattern on the outer surface of some carapace plates figured in those papers; the morphology and width/length ratio of the nuchal (plates 1a, b in Groessens-Van Dyck;^21,22^) the short first costal and the morphology of the contact area with the neurals (plate 1c, d in Groessens-Van Dyck^21^); the morphology of the other preserved costals and neurals (plate 1e–g in Groessens-Van Dyck;^21^ plate 1c in Groessens-Van Dyck^22^), and of some peripherals (plate 2e–f in Groessens-Van Dyck^21^; plate 1j–k in Groessens-Van Dyck^22^); and the arrangement of the cervical, vertebral and marginal scutes on those plates are compatible with the characters in *Laurasichersis relicta*, some of them being exclusive for this form (see diagnosis). Considering the limited number of characters provided by the hitherto known material of the taxon from Hainin, and the temporal distance between that site and the type locality of *L. relicta*, the Belgian turtle is determined as *Laurasichersis* sp. Therefore, the dispersion of Sichuanchelyidae from Asia to Europe did not occur during the upper Thanetian, but earlier, at least during the first stage after the Cretaceous-Paleogene mass extinction event, or before it.

The replacement in the European biodiversity of the terrestrial stem turtles known in the Mesozoic record of this continent by an Asian lineage (to which the only terrestrial stem turtle identified in the Cenozoic record of Laurasia belongs, the new *Laurasichersis*), was succeeded, probably at the beginning of the Eocene, by a new faunal change that involved the replacement of the stem forms by members of a more derived lineage. Thus, from the early Ypresian (early Eocene) a single lineage of terrestrial turtles is recognized in Europe, being also present in many other regions of Laurasia, which corresponds to the crown group: Testudinidae^23^. This lineage, with an ample worldwide distribution, continues to be part of the current biodiversity of Europe^24^.

## Methods

All specimens of *Laurasichersis relicta* gen. et sp. nov. studied here are deposited in the Paleontology Collection of the Muséum national d’Histoire naturelle, Paris, France (MNHN.F). In addition to the detailed comparative anatomical study of the new taxon with other stem turtles, especially with all those from the Cretaceous of Laurasia (based on personal observations, as well as in the information provided in previous papers^1,2,4,5,12–14,17–20^), the information about this form is included in the data matrix recently used for the study of the European stem turtle *Kallokibotion bajazidi*^4^. The encodings of some characters for the pleurosternids *Pleurosternon bullockii* and *Dorsetochelys typocardium* are modified (taking into account the current knowledge about the information relative to the shell of this last taxon^25^, so far not considered in that data matrix), as well as those of some of the stem turtles *Kallokibotion bajazidi*, *Sichuanchelys chowi*, ‘*Sichuanchelys*’ *palatodentata* and *Mongolochelys efremovi* (see Appendix). Following Joyce *et al*.^2^, the sauropterygian *Simosaurus gaillardoti* is not considered in the analysis. The data matrix, in which 244 characters and 119 taxa are considered, has been analyzed using TNT 1.5^26^ in order to find the most parsimonious trees (MPTs). As in the previous study in which this matrix was used^4^, a traditional search was performed, with 1,000 replications of Wagner trees (using random addition sequences), followed by tree bisection recognition (TBR) as a swapping algorithm, saving 100 trees per replication. Following the methodology previously used with the aim of solving the large polytomy that includes many stem turtles more derived than *Proterochersis robusta*, as well as several members of the crown Cryptodira^4^ (but in which *L. relicta* is also obtained as the sister taxon of *M. efremovi* within a branch of this polytomy corresponding to Sichuanchelyidae), a pruned strict tree was generated. Thus, the wild-card taxa are identified (Trees → Comparisons → Pruned Trees, with the option “listed as text” selected), with the removal of the most unstable OTUs a posteriori. Six taxa act as wild-card taxa in that analysis (*Macroclemys schmidti*, *Shachemys laosiana*, *Annemys levensis*, *Xinjiangchelys junggarensis*, “*Ordosemys* skull” and *Chengyuchelys*). A reduced consensus tree has been calculated, also using the same parameters for a TNT 1.5 traditional search, after pruning the wild-card taxa.

## Appendix

### Encoding of *Laurasichersis relicta* gen. et sp. nov. in the data matrix used by Pérez-García & Codrea^4^, based on that proposed by Joyce *et al*.^2^, and amendments in the codifications of other taxa

Encoding for *Laurasichersis relicta* gen. et sp. nov.: 27, 1; 54, 0; 56, 1; 58, 2; 59, 0; 62, 0; 65, 0; 66, 0; 68, 0; 70, 0; 71, 0; 72, 1; 77, 0; 78, 1; 79, 0; 88, 1; 90, 0; 106, 1; 109, 0; 110, 0; 111, 1; 112, 0; 113, -; 114, 0; 115, 0; 116, 0; 118, 1; 119, 1; 120, 0; 121, 0; 122, 0; 123, 0; 124, 0; 126, 1; 127, 2; 128, 1; 129, 1; 131, 0; 132, 1; 133, 0; 134, 1; 137, 1; 138, 0; 139, 1; 140, 0; 141, 0; 142, 1; 143, 1; 144, 0; 145, 0; 146, 1; 147, 0; 148, 0; 149, 0; 150, 0; 151, 0; 152, 0; 153, 0; 154, 0; 155, 0; 156, 0; 157, 1; 158, 0; 159, 0; 160, 0; 161, 0; 162, 0; 163, 0; 164, 0; 165, 0; 166, 0; 167, 1; 168, -; 197, 1; 198, 2; 199, 1; 228, 0; 229, 1; 230, 0; 231, 3; 233, 0; 235, 0; 236, 0; 237, 1; 242, 0; 243, 0; 244, 0.

New characters encoded for *Dorsetochelys typocardium*: 109, 0; 110, 0; 111, 1; 112, 1; 113, 1; 115, 0; 116, 0; 117, 0,1; 118, 1; 119, 1; 121, 0; 122, 0; 123, 0; 124, 0, 125, 2; 126, 0; 127, 2; 128, 1; 129, 1; 130, 1; 131, 1; 132, 0; 133, 0; 134, 0; 135, -; 136, -; 137, 1; 140, 0; 141, 0; 142, 0; 144, 1; 145, 0; 146, 1; 147, 0; 148, 1; 149, 0; 150, 1; 151, 0; 152, 0; 153, 0; 154, 0; 155, 0; 156, 0; 157, 1; 158, 0; 159, 0; 160, 0; 161, 0; 162, 0; 163, 0; 164, 0; 165, 0; 166, 0; 167, 0; 168, -; 233, 0; 235, 0; 242, 0; 243, 0; 244, 0.

Amended encodings for *Pleurosternon bullockii*: 117, 0 = >0,1; 125, 1 = >2; 150, 0 = >1; 154, 1 = >0; 158, 0 = >0,1.

Amended encodings for *Kallokibotion bajazidi*: 78, 0 = >1.

Amended encodings for *Mongolochelys efremovi*: 118, 0 = >1; 134, 0 = >1; 146, 0 = >?; 156, 1 = >0.

Amended encodings for ‘*Sichuanchelys*’ *palatodentata*: 118, 1 = >?; 133, 1 = >?; 146, 0,1 = >?; 156, 1 = >0.

Amended encodings for *Sichuanchelys chowi*: 156, 1 = >0.
